# Current News and Opinion

**Published:** 1883-04

**Authors:** 


### Current News and Opinion.

MARRIED.—In Philadelphia, on Thursday, April 26th, Dr. Safford G. Perry, of New York, to Miss Frances S. Thomas, the sister of Mrs. Dr. Edwin T. Darby, of Philadelphia.

It is seldom that the clinical journalist is called upon to chronicle an event which gives him greater pleasure than does the publication of the above notice. In all the crises of a man's life there is none upon which his welfare and happiness more directly depend than upon the selection of one who is to be irrevocably united to his fortunes, and to whom he must first turn for sympathy in his trials and triumphs. The union so lately celebrated seems fraught with nothing but good, for, aside from the pair whose interests are henceforth one, it brings into yet closer relationship two men, both eminent in their chosen profession, whose intimacy and friendship has been long and close. If the hearty congratulations and earnest good wishes of hundreds of professional and personal friends can avail anything, Dr. Perry's domestic hearthstone will be a haven of rest and joy, and his matrimonial bark will sail in quiet seas, wafted by none but favoring gales.

A Good Thing Well Bestowed.—At the commencement exercises of the University of Louisiana, the degree of Doctor of Medicine was conferred upon Geo. J. Friederichs, D. D. S., of New Orleans. The worthy recipient of this honor is well known to the dental profession, of which he has for many years been a prominent member. He has been a hard-working student, not alone in dental but in general pathology as well, and his utterances in our principal society meetings have ever been listened to with attention and respect. Henceforth he has an additional claim on our consideration because of his membership in the parent profession, and we are sure that his zeal will know no abatement upon this attainment of a worthy ambition.

American Medical Association.—Section on Dental and Oral Surgery.—The thirty-fourth annual session will be held in Cleveland, Ohio, commencing Tuesday, June 5th, 1883, at 11 A. M., and continuing four days.

The delegates shall receive their appointments from permanently organized State Medical Societies, and such county and district medical societies as are recognized by representation in their respective State societies, and from the Medical Department of the Army and Navy, and the Marine Hospital service of the United States.

All medical men practicing the specialty of Dental Surgery are most cordially invited to procure credentials from their local medical societies and join us at Cleveland. Railroads furnish reduced rates to all members wishing to attend.

A member desiring to read a paper before any section, should forward the paper or its title and length (not to exceed twenty minutes in reading) to the Chairman of the Committee of Arrangements, at least one month before the meeting.

Truman W. Brophy,
*Sec'y Section on Dental and Oral Surgery, Am. Med. Association.*

American Dentists in Europe.—The American Dental Society of Europe will hold its next annual meeting at Cologne, Germany, beginning on Tuesday, August 7th, 1883. Especial efforts are being put forth to make this meeting one of interest and profit. Members of the profession intending to visit Europe the coming summer are most cordially invited to be present.

W. D. Miller, Secretary, Berlin.

Dental Society of the State of New York.—The fifteenth annual meeting of the Dental Society of the State of New York will be held at Geological Hall, State street, Albany, Wednesday and Thursday, May 9th and 10th, 1883.

*Officers:*—President, L. S. Straw, Newburgh; Vice-President, Frank French, Rochester; Treasurer, A. H. Brockway, Brooklyn; Secretary, J. Edw. Line, Rochester; Correspondent, W. H. Atkinson, New York.

*Censors:*—N. W. Kingsley, Chairman, New York; S. D. French, Troy; S. B. Palmer, Syracuse; F. French, Secretary, Rochester; Wm. Jarvie, Jr., Brooklyn; W. H. Colegrove, Johnstown; A. M. Holmes, Morrisville; A. P. Southwick, Buffalo.

Members of the profession who purpose presenting themselves before the Board of Censors for examination for the diploma of this Society and the degree of "M. D. S.," should communicate their intention immediately to Dr. Frank French, Secretary of the Board, at Rochester, and report to him personally the morning of May 8, 1883, at the Delevan House, Albany. The examinations will begin at 10 A. M., and continue throughout the day, or until the list of candidates is exhausted. No examinations will be held during the sessions of the Society.

The "Whitney Memorial Prize" of $35 in cash is annually awarded to such member of this society as shall, in the opinion of the Committee on Prize Essays, offer the best paper upon some subject relating to dental science.

This prize is open to members of this society only, and by vote of the society the regularly appointed essayists may enter their papers if they choose to do so.

If the writer so elect he may withhold his name until the award is made.

All essays entered must be handed to the above committee at least ten days before the annual meeting. J. Edw. Line, Secretary.

Northern Ohio Dental Association.—The twenty-fourth annual meeting of this society will be held in the parlors of the Sloan House, Sandusky, on Tuesday and Wednesday, May 8th and 9th, beginning at 10 o'clock A. M. A cordial invitation is extended to the profession, and it is hoped and expected that there will be a large attendance.

Georgia State Dental Society.—By order of the President, and with the approval of the Executive Board, the annual meeting of this society has been changed from May to the second Monday of August, 1883, at Atlanta, Ga.

Mad River Dental Society.—The annual meeting of this society will be held at the Phillips House, Dayton, Ohio, on Tuesday, May 22, commencing at 10 o'clock A. M. A general invitation is extended to all members of the profession to be present and take part in the meeting.

The New Departure of the Association of American Medical Editors.—We have before us the circular issued by the secretary of the association. It is the culmination of his very earnest efforts to put some life and usefulness into the association. The main features of the circular are briefly as follows : The place of the next meeting will be the same as that of the American Medical Association : Cleveland, O. The time will be June 5th and 6th. Arrangements have been made so that the sessions can be held between the afternoon meetings of the sections and the evening entertainments. Dr. N. S. Davis, president of the association, will make an address on Tuesday evening, upon "The Present Status and Tendencies of the Medical Profession and Medical Journalism." On Wednesday evening, Dr. H. O. Marcy will give an address upon "Journalism Devoted to the Protection and Concentration of Medical and Surgical Science in Special Departments." Papers are also expected from Dr. Stone, of St. Paul, and Dr. Octerlony, of Louisville. The announcement is given thus early that such as are interested in these subjects, whether or not they be editors, may make such arrangements as shall permit them to take part in the discussions. It is also to be hoped that the medical editors will be out in full force. Most of them never attend the meetings of the American Medical Association, and this furnishes all an excellent special inducement to attend and make its acquaintance. We are certain that in no other way can so correct an idea be obtained of the medical profession of the entire United States ; of its excellencies and its defects, and suggestions as to the ways by which the one may be cultivated and the other corrected.—*Detroit Lancet.*

